# ADS-HCSpark: A scalable HaplotypeCaller leveraging adaptive data segmentation to accelerate variant calling on Spark

**DOI:** 10.1186/s12859-019-2665-0

**Published:** 2019-02-14

**Authors:** Anghong Xiao, Zongze Wu, Shoubin Dong

**Affiliations:** Communication & Computer Network Lab of Guangdong, School of Computer Science & Engineering, South China University of Technology, Wushan Road, Guangzhou, 510641 China

**Keywords:** Variant calling, Spark, Adaptive data segmentation, Hadoop-BAM

## Abstract

**Background:**

The advance of next generation sequencing enables higher throughput with lower price, and as the basic of high-throughput sequencing data analysis, variant calling is widely used in disease research, clinical treatment and medicine research. However, current mainstream variant caller tools have a serious problem of computation bottlenecks, resulting in some long tail tasks when performing on large datasets. This prevents high scalability on clusters of multi-node and multi-core, and leads to long runtime and inefficient usage of computing resources. Thus, a high scalable tool which could run in distributed environment will be highly useful to accelerate variant calling on large scale genome data.

**Results:**

In this paper, we present ADS-HCSpark, a scalable tool for variant calling based on Apache Spark framework. ADS-HCSpark accelerates the process of variant calling by implementing the parallelization of mainstream GATK HaplotypeCaller algorithm on multi-core and multi-node. Aiming at solving the problem of computation skew in HaplotypeCaller, a parallel strategy of adaptive data segmentation is proposed and a variant calling algorithm based on adaptive data segmentation is implemented, which achieves good scalability on both single-node and multi-node. For the requirement that adjacent data blocks should have overlapped boundaries, Hadoop-BAM library is customized to implement partitioning BAM file into overlapped blocks, further improving the accuracy of variant calling.

**Conclusions:**

ADS-HCSpark is a scalable tool to achieve variant calling based on Apache Spark framework, implementing the parallelization of GATK HaplotypeCaller algorithm. ADS-HCSpark is evaluated on our cluster and in the case of best performance that could be achieved in this experimental platform, ADS-HCSpark is 74% faster than GATK3.8 HaplotypeCaller on single-node experiments, 57% faster than GATK4.0 HaplotypeCallerSpark and 27% faster than SparkGA on multi-node experiments, with better scalability and the accuracy of over 99%. The source code of ADS-HCSpark is publicly available at https://github.com/SCUT-CCNL/ADS-HCSpark.git.

**Electronic supplementary material:**

The online version of this article (10.1186/s12859-019-2665-0) contains supplementary material, which is available to authorized users.

## Background

In the past decade, next generation sequencing (NGS) technology has made great progress and personal genome sequencing has also been widely used in human disease research, clinical treatment and new drug research [1]. In the genome analysis process, variant calling is significant step to discover and obtain variants relative to reference genome, which is also the basis for subsequent analysis. GATK [2, 3] from the Broad Institute is one of the mainstream NGS genome data analysis toolkits, which focuses on processing variant discovery and genotyping of both exomes and whole genomes generated by Illumina sequencing machines. In GATK, HaplotypeCaller is the most prevalent variant calling approach applied to the discovery of short variant in germ cells. Its capability of calling SNPs and indels simultaneously through local de-novo assembly of haplotypes in an active region, which makes the HaplotypeCaller much better at calling indels [4] than other position-based callers such as Samtools [5] and GATK UnifiedGenotyper.

However, with the dramatic increasing of genome data, it will take a long time to perform variant calling. GATK HaplotypeCaller runs on a single node with serious scalability bottleneck, which leads to inefficient use of the computing resources, especially dealing with large scale genome data. The calculation of HaplotypeCaller is complex, mainly including four steps: identifying active regions, local reassembly, likelihood calculation and assigning genotypes. In the study [6], the time consumption of various parts of HaplotypeCaller is counted as shown in Table 1. Among them, “Assembly” is the second step, local reassembly of HaplotypeCaller, and “PairHMM” is the third step, likelihood calculation. “Traversal + Genotyping” includes traversing alignment sequence data, identifying active regions and assigning genotypes. It could be seen that the most time-consuming step in HaplotypeCaller is the calculation of PairHMM, which takes up to 70% of the total time.

**Table 1:** The runtime for each step of HaplotypeCaller [6]

Stage	Runtime	Percentage
Assembly	2598 s	13%
PairHMM	14,225 s	70%
Traversal + Genotyping	3379 s	17%

It is reported [7] that there is a serious problem of computation skew in HaplotypeCaller, meaning that though the size of input file is the same, the running time of variant calling is still significantly different. This is mainly caused by some difference in sequence data. This problem poses a great challenge to the parallelization of HaplotypeCaller, which easily causes long tail tasks and leads to poor scalability.

Recently, cloud computing and big data technology have become increasingly popular. A couple distributed frameworks such as Hadoop and Apache Spark [8] have emerged to provide excellent solutions for addressing the scalability problem of variant calling. Hadoop/Spark are big data frameworks that provide highly parallel distributed computing environment using multiple ordinary machines to store and analyze large datasets faster and more efficiently. Spark could achieve higher performance than Hadoop due to its memory-based computing. A growing number of genome analysis tools based on distributed framework have been proposed [9, 10]. GATK-Queue [11] is an extension of GATK that uses Sun Grid Engine to run tasks in a distributed cluster in Scatter-gather mode, but its parallel approach is command-based, whose task segmentation is large and cannot be fine-grained. Halvade [12] implements genome analysis process using Hadoop MapReduce based approach, in which the variant calling tasks are divided by chromosome. This division is likely to cause load imbalance due to the obvious difference in the length of human chromosomes. Churchill [13] is a tightly-integrated DNA analysis pipeline and can implement variant calling using FreeBayes [14] or HaplotypeCalller. Its parallel strategy is to divide the data by the same size and perform variant calling in parallel on each segment, which can overcome the load imbalance caused by uneven chromosome length to some extent. Nevertheless, it does not solve the problem of computation skew. SparkGA [15] is a parallel implementation of a genome analysis pipeline based on Spark, in which the parallel strategy of the variant calling is relatively simple and does not consider the overlap of adjacent blocks. In addition, the official version of the latest GATK4.0 [16] was released and many analysis tools are redeveloped based on Spark framework. GATK4.0’s multi-node variant caller HaplotypeCallerSpark also implements parallelization of variant calling on multi-node and multi-core based on Spark framework, but it has a high demand for computing resources. When HaplotypeCallerSpark runs on large scale datasets, huge memory overhead and time-consuming shuffle operators have become a bottleneck.

Thus, in order to accelerate variant calling on large scale genome data, a high scalable tool which could run in distributed environment is demanded. In this paper, we proposed ADS-HCSpark, a scalable tool to accelerate the stage of variant calling based on Spark framework, which implements the parallelization of GATK3.8 HaplotypeCaller algorithm on the cluster of multi-core and multi-node. The source code and usage document of ADS-HCSpark are respectively described in the Additional files 1 and 2. The main contributions of our work are as follows:

• A parallel strategy of adaptive data segmentation is proposed and a variant calling algorithm based on adaptive data segmentation (ADS-HC) is implemented to address the problem of computation skew in HaplotypeCaller.

• For the requirement that adjacent data blocks should have overlapped boundaries, Hadoop-BAM library is customized to implement partitioning BAM file into overlapped blocks, improving the accuracy of variant calling.

## Implementation

### Overview of ADS-HCSpark

Current variant caller is relatively inefficient which takes lots of time to perform variant calling, and ADS-HCSpark is proposed to achieve parallelization of HaplotypeCaller to accelerate the process of variant calling. In the distributed environment, the input BAM file is usually segmented into equal-sized original data blocks for parallel processing by default on HDFS. Due to the computation skew of HaplotypeCaller, the processing of some data blocks may take a very long time. To address the problem of computation skew in HaplotypeCaller, we propose a parallel strategy of adaptive data segmentation. Adaptive data segmentation aims to divide the original time-consuming blocks into multiple new blocks and keep the rest in their original partitions, to ensure all the blocks would be processed in almost same execution time ideally. Due to the scheduling mechanism (that the next task in the task queue is performed when there is an idle core) of Spark framework, if the number of data blocks is reasonable and there is no obvious long tail task, the whole program is generally load balanced.

**Fig. 1:**
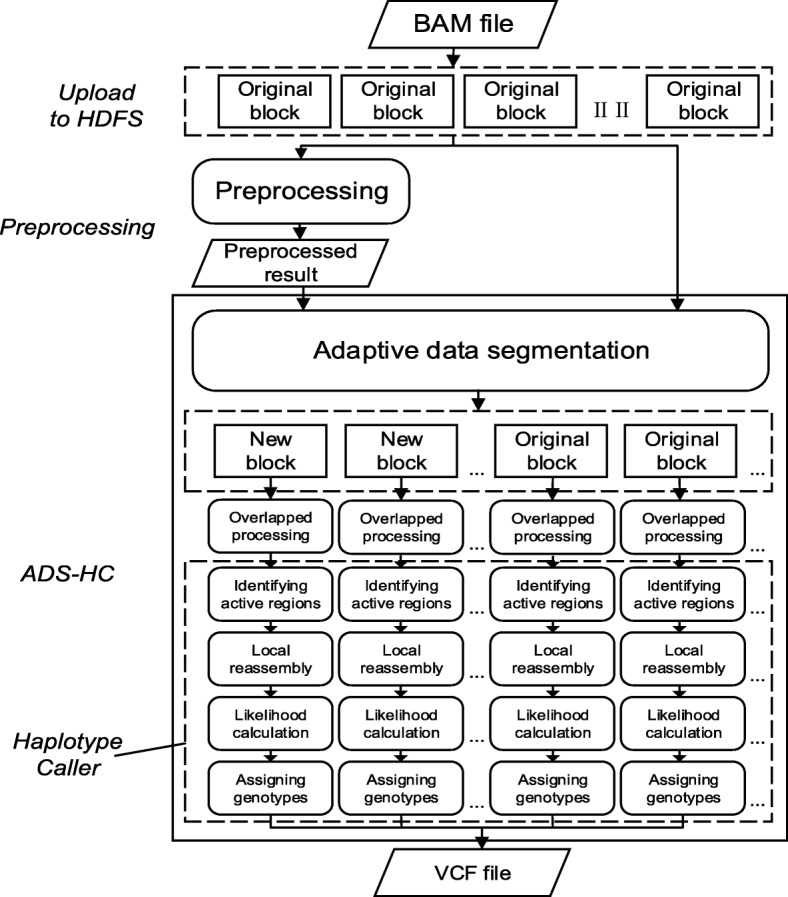
The flow-process diagram of ADS-HCSpark. The figure shows the execution flow of ADS-HCSpark. First the BAM file needs to be uploaded to HDFS. ADS-HCSpark includes two parts: the data preprocessing and the variant calling based on adaptive data segmentation (ADS-HC). ADS-HC includes targeted data partitioning, overlapped processing, variant calling and output merge. Among them, variant calling consists of four main steps of GATK HaplotypeCaller: identifying active regions, local reassembly, likelihood calculation and assigning genotypes

Therefore, our target is to find the original time-consuming data blocks and apply appropriate segmentation. According to the Table 1, the third step PairHMM takes up most of running time, so if runtime of PairHMM could be estimated, the runtime of the data block also could be estimated roughly. The time complexity of PairHMM is O(N × M × R × H) [6], in which N is the number of reads, M is the number of candidate haplotypes, R is total length of reads and H is total length of candidate haplotypes. In order to estimate the time consumption of PairHMM, the first two steps of HaplotypeCaller have to be performed, which will increase the time by at least 20%. Accurately estimating the running time of variant calling for a data block is complex and time-consuming, so for further simplifying the calculation, in ADS-HCSpark, above task is converted to use the sequence features to determine whether it takes long execution time to process the data block. The parallel strategy of adaptive data segmentation is implemented by combining the file partitioning mechanism of HDFS and the scheduling mechanism of Spark based on the sequence features of input file. The flow-process diagram of ADS-HCSpark is shown in Fig. 1.

ADS-HCSpark is divided into two parts: the data preprocessing to mining sequence features of input file and the variant calling based on adaptive data segmentation (ADS-HC). ADS-HC includes targeted data partitioning, overlapped processing, variant calling and output merge. Among them, variant calling consists of four main steps of GATK HaplotypeCaller: identifying active regions, local reassembly, likelihood calculation and assigning genotypes.

### Data preprocessing

According to the previous analysis of HaplotypeCaller, it can be inferred that accurately predicting the execution time of variant calling for a data block is quite complex and time-consuming. In order to simplify the calculation, the above task is converted to use the sequence features to determine whether it takes long execution time to process the data block in ADS-HCSpark. As described above, the time complexity of most time-consuming part PairHMM of HaplotypeCaller is O(N × M × R × H), which means that the execution time is related with sequence and candidate haplotypes. However, to obtain features of candidate haplotypes, the first two steps of HaplotypeCaller have to be performed, which will increase the runtime of preprocessing stage. To simplify calculations and reduce extra time, we select relevant sequence features which could be counted and retrieved by scanning the original input file once. The relevant sequence features are demonstrated in Table 2.

**Table 2:** Relevant sequence features obtained in the preprocessing stage

Sequence features	Comment
Index ID	Index number of data block
Interval	Interval length of all the alignment sequence in the data block
Record Num	Number of all the alignment sequence in the data block
CIGAR_I	Sum of the insertion lengths of all the alignment sequence in the data block
CIGAR_D	Sum of the deletion lengths of all the alignment sequence in the data block

Above sequence features intuitively reflect the characteristics and variation situation of sequence. Their differences could affect the execution time of variant calling. In order to obtain these sequence features, the data preprocessing is required. First of all, the input BAM file should be uploaded to HDFS where the BAM file is partitioned into several fix size data blocks (eg.128 MB by default). In the data preprocessing stage, ADS-HCSpark reads each block in parallel and counts sequence features of each block according to the corresponding field of every record in the block. Among sequence features, Interval and RecordNum can be obtained by separately counting the number of bases and the number of records in the data block. CIGAR_I and CIGAR_R could be calculated from the field CIGAR of every record in the block. Finally, all the sequence features are saved into the preprocessing result file. The algorithm description and specific implementation details are described in the Additional file 3.

### Adaptive data segmentation

In the variant calling stage based on adaptive data segmentation, we need to predict and divide the time-consuming data blocks according to the sequence features. Therefore, the first step is to determine the number of data blocks to be segmented and how to select these data blocks.

In order to determine which block needs to be segmented, we select an input BAM file to analyze the execution time of each original block. The variant caller HaplotypeCaller is separately executed on every original blocks of the BAM file on HDFS and their respective running time is recorded. Then data blocks are sorted by their execution time from high to low and it could be concluded that the running time of top n% of data blocks is obviously longer than that of others by statistics (The value of n will be discussed in the later experimental part). Thus, we consider this top n% of data blocks as the long time-consuming blocks and our target is to predict and segment them.

The sequence features obtained in preprocessing stage could reflect the computational complexity of variant calling to some extent. Generally, the reads could be mapped to the reference sequence, but when there are more insertions and deletions in the alignment reads, more candidate haplotypes are easily generated, which leads to more subsequent time-consuming analysis. CIGAR_I and CIGAR_D in the preprocessed result file reflect the approximate number of inserted and missing segments in the data block. Usually, the distribution of alignment sequences is relatively uniform, but when they are too concentrated or sparse, the variation situation is more complicated and more calculations are required. In the sequence features, this is reflected that the range of the site covering the chromosome within the data block is too short or long. In general, the number of alignment sequence in every data block is equivalent. When the number of alignment sequence in some data blocks is significantly less than that in others, it is owing to the effect of the filters of HaplotypeCaller, indicating that part of alignment sequence in these data blocks are unreliable and need to be filtered. The alignment sequence situation in this region may be more complex and it is likely to require more calculations to execute variant calling, resulting in a time-consuming increase.

Based on the above analysis, our segmentation target is the top n% of the most time-consuming data blocks and they are predicted according to the following four rules. The parameters of rules and specific segmentation ratio will be discussed in the later experimental part.

• Top m% of the data blocks sorted by Interval from low to high.

• Top k% of the data blocks sorted by Interval from high to low.

• Top s% of the data blocks sorted by RecordNum from low to high.

• Top r% of the data blocks sorted by (CIGAR_I + CIGAR_D) from high to low.

These four rules correspond to several time-consuming situations analyzed above. The first two rules filter out the data blocks in which alignment sequence distribution is too concentrated or too sparse. The third rule filters out those data blocks in which the number of alignment reads is significantly less than that in others and the last rule filters out those blocks in which there are more insertions and deletions. These filtered data blocks are potential blocks that cause a long time for variant calling. In order to find time-consuming data blocks as much as possible, we do not consider priorities among above rules and as long as sequence features satisfy any rule, this data block is predicted to be time-consuming.

In the process of adaptive data segmentation, ADS-HCSpark first reads the sequence features of each original data block from the preprocessing result file and then all the original data blocks are sorted according to the requirements of four rules mentioned above. For the data blocks that satisfy any one rule, they are considered as the time-consuming block and their index numbers are stored in a collection. These blocks will be segmented into multiple new blocks which are set to high priority to execute variant calling. Other blocks that are not in the collection are not segmented and set to standard execution priority. After completing adaptive data segmentation, all the data blocks will be sorted by their execution priority, thereby ensuring that time-consuming blocks will be processed firstly. The algorithm description for computing the index number of data block to be segmented and segmenting data blocks are respectively described in the Additional files 4 and 5.

### Customized Hadoop-BAM for overlapped blocks

After adaptive data segmentation, new data blocks will be read in parallel for processing. Since the variant calling of one site is associated with the alignment information of the sites in the vicinity, simple partitioning strategy by data block may lead to unreliable results. For higher accuracy of variant calling, ADS-HCSpark adopts an approach to partition data blocks with overlapped boundaries of adjacent data block. Hadoop-BAM [17] is a library commonly used to read BAM files in parallel by Spark and Hadoop, but it cannot achieve overlapped processing between adjacent data blocks, which needs to be customized. Thus, we improved the Hadoop-BAM library to implement partitioning BAM file into overlapped blocks. In ADS-HCSpark, the size of overlapped boundaries of adjacent data blocks is set to the parameter *overlapSize* and different values of this parameter will affect the result of subsequent variant calling. The experiment is conducted to evaluate it in detail in the later chapter. In the process of partition of BAM file with overlapped blocks, all the data block information of the BAM file is obtained firstly and then data blocks are sorted according to the block number to ensure that data blocks are order. Then the program traverses all the data blocks and except for the last data block, the rest need to be extended the size of overlapped boundary. After overlapped processing, the boundary of two adjacent data blocks are the same. The size of overlapped boundary is up to the parameter *overlapSize*. Finally, the program returns all the overlapped blocks. The algorithm description for acquiring overlapped data blocks is described in the Additional file 6.

### Algorithm framework of ADS-HCSpark

In the step of variant calling, ADS-HCSpark uses the main algorithm of HaplotypeCaller to discover and obtain variants. After adaptive data segmentation and overlapped processing, ADS-HCSpark performs operations such as identifying active regions, local reassembly, likelihood calculation and assigning genotypes for all the alignment data in each data blocks in parallel. Finally, all the variants discovered are merged and output into a VCF file.

**Fig. 2:**
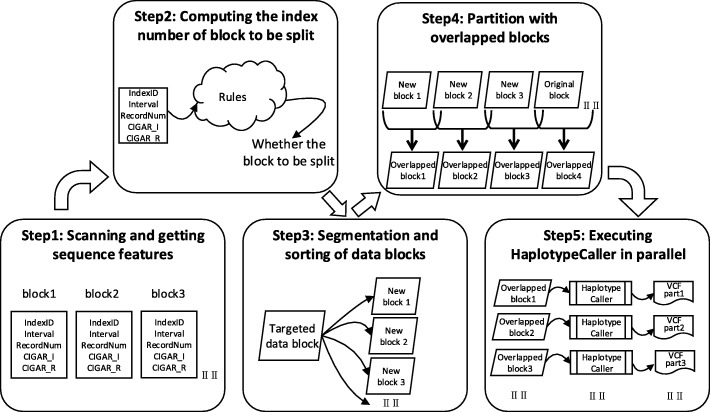
Algorithm framework diagram of ADS-HCSpark. The figure shows the entire algorithm framework of ADS-HCSpark. In the preprocessing, the program scans the input BAM file to obtain the sequence features of each original block. According to the preprocessing result and the rules mentioned above, data blocks to be split are predicted and segmented. Then overlapped blocks are read in parallel by customized Hadoop-BAM library and finally variant calling is executed on them

Combining all the above steps, the entire algorithm framework of ADS-HCSpark is illustrated in Fig. 2. In the preprocessing, the program scans the input BAM file to obtain the sequence features of each original block. According to the preprocessing result and the rules mentioned above, data blocks to be split are predicted and segmented. Then overlapped blocks are read in parallel by customized Hadoop-BAM and finally variant calling is executed on them.

## Results

### Experiment setup

ADS-HCSpark is evaluated on our cluster with 6 nodes. Each node is equipped with two E5–2670 CPU (2.6GHz, 8 cores) with 64 GB memory. The network is 1 GigE. Spark version is 2.2.0. Scala version is 2.11.8. The datasets used in the experiments are from the reference [18] and the human genome data are selected. The datasets are described in detail in the Table 3. Some program execution scripts and dataset details in the experiments are respectively described in the Additional files 7 and 8.

**Table 3:** Experimental datasets

Dataset	Genome	File format	Coverage depth	File size	Default number of data blocks
D1	NA12878	BAM	14x	67.7GB	543
D2	NA12878	BAM	28x	128.5GB	1028
D3	NA18507	BAM	11x	59.3GB	475
D4	NA12878	BAM	60x	250.15GB	2002

### Parameters of adaptive segmentation

**Fig. 3:**
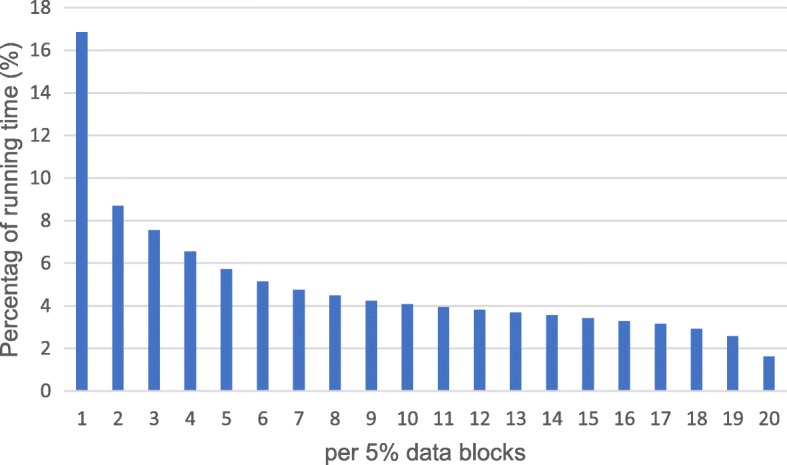
The percentage of running time per 5% data blocks after all the data blocks are sorted. The HaplotypeCaller algorithm is separately executed on every original data blocks of dataset D1 and their respective running time is recorded. Then data blocks are sorted by execution time from high to low and the percentage of time consumption for per 5% data blocks is counted, as is shown in the figure

As mentioned above, our segmentation target is the top n% of the most time-consuming data blocks. In order to determine the value of n, the HaplotypeCaller algorithm is separately executed on every data block of the dataset D1 and their respective running time is recorded. Then data blocks are sorted by execution time from high to low and the percentage of time consumption for per 5% data blocks is counted, as is shown in Fig. 3. It could be clearly found that the top 5% of the data blocks are up to 16.9% of the total running time and obviously higher than the latter. Thus, we consider this top 5% of data blocks as the long time-consuming blocks and our target is to predict and segment them.

To determine the parameters of predict rules, the specific segmentation ratio is adjusted on the dataset D1 and verified on the dataset D2, D3 and D4. The parameters chosen should allow our approach to find as many time-consuming raw data blocks as possible. For the parameters of four rules, when we set m% = 5%, k% = 7%, s% = 5%, and r% = 7%, the detailed situation of the segmentation indicators in each dataset is shown in Table 4. Segmenting precision is defined as: $\mathrm{P}=\frac{TP}{N}$, and segmenting recall is defined as: $\mathrm{R}=\frac{TP}{M}$. TP is the number of true time-consuming data blocks in the predicted data blocks. N is the number of the predicted data blocks. M is the target number of time-consuming data blocks in the original data blocks. From Table 4, the recall rates of segmenting on four datasets are high even reaching 100%, which means that our solution could find most or even all of the top 5% of the most time-consuming data blocks. This is the reason why adaptive data segmentation can solve the problem of computation skew. As for segmenting precision, it is maintained at approximately 33%, which indicates that some of the predicted data blocks are not time-consuming, but segmenting some non-target data blocks does not affect the final running time much. Because the problem of computation skew is mainly caused by time-consuming data blocks, as long as most of time-consuming blocks are included in our predicted blocks (meaning high recall rate), long tail tasks could be effectively avoided. Thus, we give priority to a high recall rate while allowing a certain precision ratio to be sacrificed.

**Table 4:** The detailed situation of the segmentation indicators in each dataset

Dataset	D1	D2	D3	D4
BAM file size	67.8GB	128.5GB	59.3GB	250.15GB
Default number of data blocks	543	1028	475	2002
Target number of segmentations (5%)	28	52	24	102
Actual number of segmentations	84	147	76	303
Actual proportion of segmenting	15.47%	14.3%	16%	15.13%
Number of matching blocks	27	52	24	101
Segmenting Precision	32.14%	35.37%	31.58%	33.33%
Segmenting Recall	96.43%	100%	100%	99.02%

### Impact of overlapped boundaries on the variant calling accuracy

In ADS-HCSpark, the size of overlapped boundaries of adjacent data blocks is set to the parameter *overlapSize* and different values of this parameter will affect the accuracy of variant calling. The following experiments were performed to evaluate the accuracy of ADS-HCSpark under different size of overlapped boundaries of adjacent blocks. The accuracy is evaluated by comparing the variants detected by ADS-HCSpark with the results of GATK3.8 HaplotypeCaller as a baseline. The experimental result is shown in Table 5. Even though there are no overlapped boundaries of adjacent blocks, ADS-HCSpark could reach a high accuracy with over 99.9%. When there are overlapped boundaries of adjacent blocks, the accuracy of ADS-HCSpark is generally higher than that without overlapped boundaries, which explains that overlapped boundaries could maintain the integrity of variant calling. Simultaneously, overlapped boundaries of different sizes have a slight effect on the accuracy and overlapped boundaries are too small to completely cover the detection of the edges. When the size of overlapped boundaries of adjacent blocks is set to 512 KB, ADS-HCSpark achieves the highest accuracy and the accuracy tends to be stable when continuing to increase the size of overlapped area. Thus, the parameter *overlapSize* is set to 512 KB.

**Table 5:** Accuracy of ADS-HCSpark in different sized overlapped boundaries

Overlap Size	D1	D2	D3	D4
0 KB	99.9828%	99.9678%	99.9887%	99.9842%
64 KB	99.9829%	99.9686%	99.9896%	99.9844%
128 KB	99.9831%	99.9691%	99.9891%	99.9847%
256 KB	99.9832%	99.9698%	99.9891%	99.9851%
512 KB	99.9835%	99.9698%	99.9893%	99.9856%
1024 KB	99.9835%	99.9698%	99.9893%	99.9856%

### Performance analysis

#### Data preprocessing

**Fig. 4:**
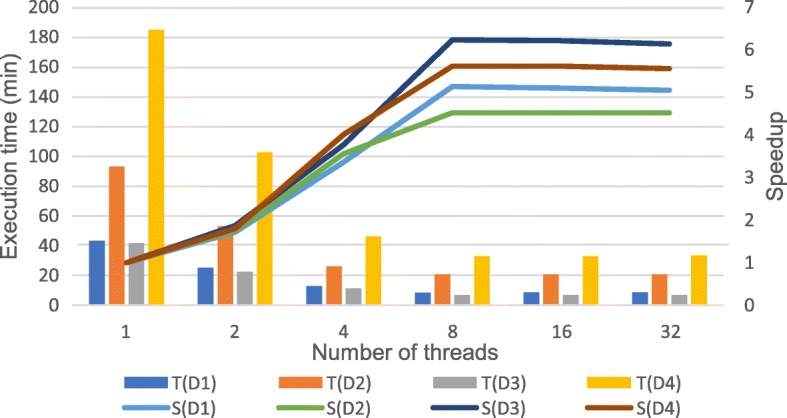
Execution time and speedup of preprocessing with different threads. The figure shows the execution time and speedup of data preprocessing on four datasets. T(D1), T(D2), T(D3), T(D4) represent the execution time of preprocessing on dataset D1, D2, D3, D4 and S(D1), S(D2), S(D3), S(D4) represent the speedup of preprocessing on dataset D1, D2, D3, D4

**Fig. 5:**
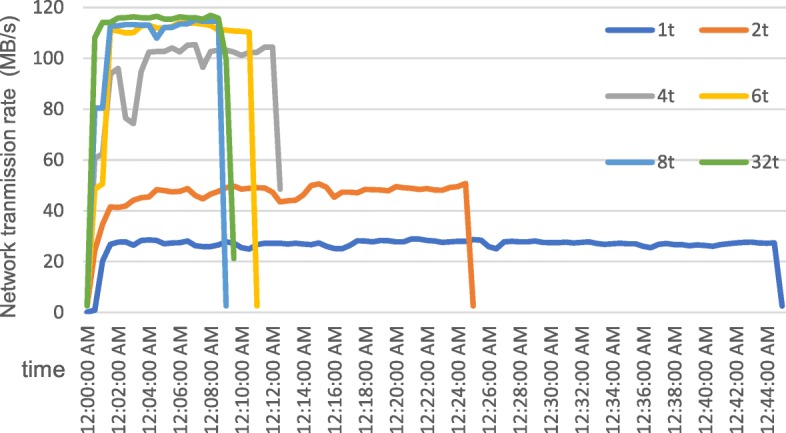
Network transmission rate of preprocessing with different threads on D1. The figure shows the comparison of network transmission rate for different threads on dataset D1. Curves of different colors indicate different threads. 1 t represents 1 thread, 2 t represents 2 threads and so on

To analyze the performance of data preprocessing, the experiment was conducted on one node with different threads. The execution time and speedup of data preprocessing on four datasets are illustrated in Fig. 4. In the figure, T(D1), T(D2), T(D3), T(D4) represent the execution time of preprocessing on dataset D1, D2, D3, D4 and S(D1), S(D2), S(D3), S(D4) represent the speedup of preprocessing on dataset D1, D2, D3, D4. Speedup is defined as: $\mathrm{S}=\frac{T_p}{T_s}$ . *T*_*p*_ represents the execution time to serially perform the algorithm and *T*_*s*_ represents the execution time to parallelly perform the algorithm on p processors. As the number of threads increases, the running time of preprocessing decreases and the speedup ratio is on the rise. When the number of threads exceeds 8, the speedup remains stable or drops slightly, which indicates that there is a bottleneck in the scalability of the preprocessing step. Figure 5 shows the comparison of network transmission rates for different threads (1 t represents 1 thread in the figure) on dataset D1, which could be found that the bottleneck of the preprocessing step is the network bandwidth. The theoretical network transmission rate of Gigabit Ethernet is 120 MB/s. When executing preprocessing with 8 threads, the network transmission is already close to the bandwidth limit. Continuing to allocate more threads brings a lower promotion of performance and even may lead to performance degradation due to excessive threads competing for network resources. Thus, the optimal number of threads for data preprocessing step is 8 in a single-node and Gigabit Ethernet environment. In a multi-node cluster, the optimal number of threads in this step is 8 threads per node.

#### Adaptive data segmentation and scalability analysis

ADS-HC needs to segment the target data blocks and the different granularity of data segmentation will also affect the running time of ADS-HC. The granularity is the number of new data blocks divided from the time-consuming block. The following experiments were conducted to evaluate the impact of various granularity of data segmentation. The running time of different fine-grained segmenting numbers on a single node with 32 threads and a 6-node cluster with 192 threads is shown in Table 6.

**Table 6:** ADS-HC running time with different granularity of data segmentation

Execution time (min)	only sorted	2 blocks + sorted	4 blocks + sorted	6 blocks + sorted	8 blocks + sorted	0 blocks + no sorted
D1 (1 node)	73.89	75.30	75.76	76.42	77.47	85.09
D2 (1 node)	100.14	101.05	103.79	105.12	103.44	106.68
D3 (1 node)	71.45	71.43	72.67	73.30	75.11	80.67
D4 (1 node)	160.59	161.40	164.32	165.93	168.43	182.85
D1 (6 nodes)	27.32	20.34	19.46	18.41	18.49	33.92
D2 (6 nodes)	33.02	28.95	27.15	26.01	26.22	40.32
D3 (6 nodes)	26.21	20.96	19.31	19.33	20.65	33.14
D4 (6 nodes)	47.38	44.42	43.75	43.22	43.22	62.13

In Table 6, “only sorted” strategy represents that after preprocessing, only data blocks are sorted by processing priority from high to low and the time-consuming data blocks are directly processed without being segmented. “n blocks + sorted” strategy means that every target data block is equally segmented to n new blocks after preprocessing and they are set to higher processing priority. Then data blocks are sorted by processing priority from high to low. The last column “0 blocks + no sorted” strategy is the control group in which the BAM file is segmented and processed by default Spark framework without any preprocessing.

In the experiment on a single node, running time of “only sorted” strategy is usually shorter than others. This is because all four datasets are quite large and their default numbers of partitions are a lot, while the degree of parallelism (execution threads) of one node is low. In this case, each thread needs to execute more tasks, so long tail tasks could be avoided by prioritizing time-consuming blocks even without fine-grained segmentation. Furthermore, excessive blocks will lead to extra scheduling overhead. Therefore, with more default blocks and lower degree of parallelism, “only sorted” strategy can achieve better results. However, when ADS-HC runs on a cluster with 6 nodes, the degree of parallelism is much more than that of one single node, so it needs to segment the default data blocks properly to avoid long tail tasks. The running time of “6 blocks + sorted” strategy is shorter than that of others. Too few segments could not avoid long tail tasks and excessive segments will cause extra overhead. Summarizing above experimental results, compared to the default blocking mode, the adaptive data segmentation strategy could effectively predict and segment the time-consuming data blocks, thus avoiding long tail tasks and addressing the problem of computation skew.

**Fig. 6:**
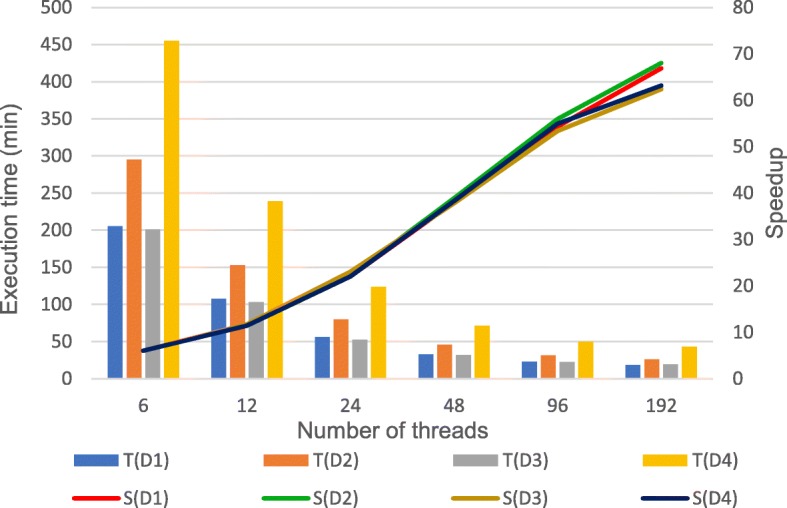
Execution time and speedup of ADS-HC with different threads on 6 nodes. The figure shows that the execution time and the corresponding speedup of ADS-HC with different threads on a 6-node cluster. T(D1), T(D2), T(D3), T(D4) represent the running time on dataset D1, D2, D3, D4 and S(D1), S(D2), S(D3), S(D4) represent the speedup ratio on dataset D1, D2, D3, D4

The scalability of ADS-HC is evaluated on a 6-node cluster with different threads using the “6 blocks + sorted” strategy. The running time and the corresponding speedup on four datasets are illustrated in Fig. 6. In the figure, T(D1), T(D2), T(D3), T(D4) represent the execution time on dataset D1, D2, D3, D4 and S(D1), S(D2), S(D3), S(D4) represent the speedup ratio on dataset D1, D2, D3, D4. From the experimental results, as the number of threads increases, the execution time decreases and the speedup rate increases. Particularly, when the number of threads increases from 1 to 96, ADS-HC achieves good scalability, and the speedup rate linearly increases, approximately. While the number of threads exceeds 96, the speedup ratio increases slowly, because the average number of threads used per node is more than 16 at this time. Although each node could support up to 32 threads with 32 logical cores, there are only 16 physical cores. In these datasets, D4 is the completed NA12878 dataset with the coverage depth of 60x. From the experimental results, ADS-HCSpark achieves good scalability for datasets of different size and coverage depth, which proves that it could be used to execute variant calling on large scale datasets.

### Comparison with GATK and SparkGA

#### Multiple threads on single node

**Fig. 7:**
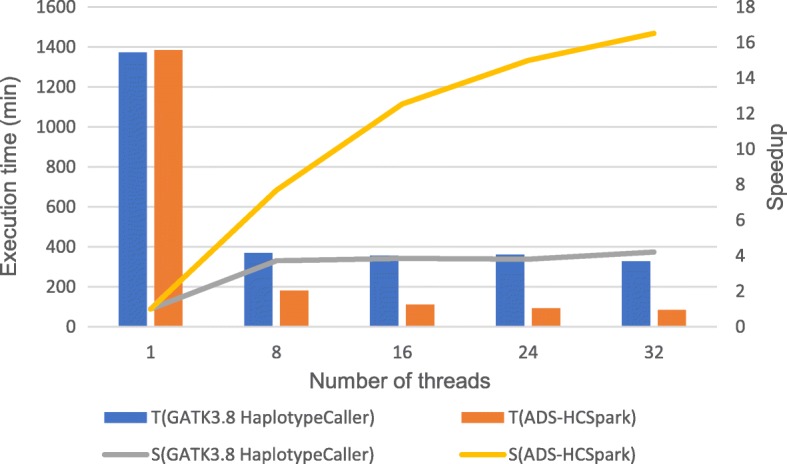
Comparison of execution time and speedup on a single node. The figure shows the comparison of execution time and speedup between GATK3.8 HaplotypeCaller and ADS-HCSpark on a single node with different threads. T (GATK3.8 HaplotypeCaller), T (ADS-HCSpark) represent the execution time of GATK3.8 HaplotypeCaller and ADS-HCSpark. S (GATK3.8 HaplotypeCaller), S (ADS-HCSpark) represent the speedup of GATK3.8 HaplotypeCaller and ADS-HCSpark

GATK HaplotypeCalller is the benchmark of variant calling tool, which supports multithreading on single node. Our ADS-HCSpark is also implemented based on GATK3.8 HaplotypeCaller. In the previous analysis, the time-consuming characteristics of four datasets are similar, so we take dataset D1 as an example to compare the execution time and scalability of ADS-HCSpark with that of GATK3.8 HaplotypeCaller on a single node. HaplotypeCaller includes two steps: building index and variant calling, while ADS-HCSpark also consists of two parts: data preprocessing and ADS-HC. The experimental result is shown in Table 7, and corresponding diagram is illustrated in Fig. 7. In the figure, T (GATK3.8 HaplotypeCaller), T (ADS-HCSpark) represent the execution time of GATK3.8 HaplotypeCaller and ADS-HCSpark. S (GATK3.8 HaplotypeCaller), S (ADS-HCSpark) represent the speedup of GATK3.8 HaplotypeCaller and ADS-HCSpark. In case of full load with 32 threads, where both tools achieve optimal performance, the running time of ADS-HCSpark is reduced by 74.33% compared to GATK3.8 HaplotypeCalller. After GATK3.8 HaplotypeCaller reaches 8 threads, the speedup remains around 4, while the speedup ratio of ADS-HCSpark continues to increase, eventually reaching 16.5, which is more scalable than GATK3.8 HaplotypeCaller. The CPU utilization of GATK3.8 HaplotypeCaller is lower than that of ADS-HCSpark, because in GATK3.8 HaplotypeCaller, data are serially read and processed firstly, consuming too much time and easily causing waiting among threads. Conversely, ADS-HCSpark uses customized Hadoop-BAM to read alignment data in parallel, achieving high CPU utilization.

#### Multiple threads on multiple nodes

**Fig. 8:**
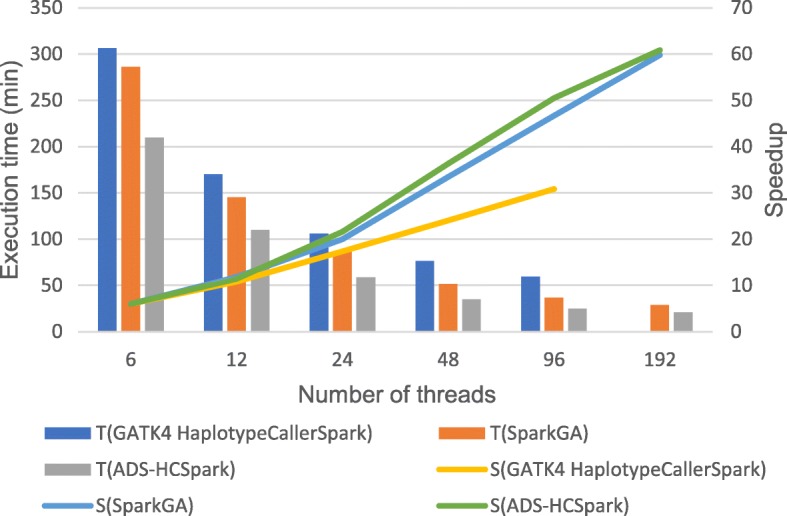
Comparison of execution time and speedup on 6 nodes. The figure shows the comparison of execution time and speedup with different threads on a 6-node cluster among three tools: GATK4.0 HaplotypeCallerSpark, SparkGA and ADS-HCSpark. T (GATK4 HaplotypeCallerSpark), T (SparkGA), T (ADS-HCSpark) represent the execution time of GATK4.0 HaplotypeCallerSpark, SparkGA and ADS-HCSpark. S (GATK4 HaplotypeCallerSpark), S (SparkGA), S (ADS-HCSpark) represent the speedup of GATK4.0 HaplotypeCallerSpark, SparkGA and ADS-HCSpark

GATK4.0 is a toolkit developed by Broad Institute based on Spark framework and HaplotypeCallerSpark is its variant calling tool, which could run on a multi-node cluster. SparkGA is also a high-performance genome analysis toolkit based on Spark framework. The experiments were conducted to compare both running time and scalability among these three tools on a cluster with 6 nodes on dataset D1. ADS-HCSpark includes data preprocessing and variant calling, while GATK4 HaplotypeCallerSpark and SparkGA only includes variant calling stage. Table 8 describes the running time of them with different threads and the corresponding diagram is illustrated in Fig. 8. In the figure, T (GATK4 HaplotypeCallerSpark), T (SparkGA), T (ADS-HCSpark) represent the execution time of GATK4.0 HaplotypeCallerSpark, SparkGA and ADS-HCSpark. S (GATK4 HaplotypeCallerSpark), S (SparkGA), S (ADS-HCSpark) represent the speedup of GATK4.0 HaplotypeCallerSpark, SparkGA and ADS-HCSpark. Since GATK4 HaplotypeCallerSpark consumes a large amount of memory, this experimental platform cannot support HaplotypeCallerSpark running on 6 nodes over 96 threads. With 96 threads, ADS-HCSpark is 57.69% faster than GATK4 HaplotypeCallerSpark. Besides, its speedup is low and only reaches around 30 at the end. The speedup of ADS-HCSpark continues to increase and eventually reaches 60. Therefore, the scalability of ADS-HCSpark on 6 nodes is far better than that of GATK4 HaplotypeCallerSpark. As for the comparison between SparkGA and ADS-HCSpark, in case of full load with 192 threads, ADS-HCSpark is 27.91% faster than SparkGA and it is similar in the trend of their speedup ratios. Since the adaptive data segmentation, ADS-HCSpark effectively avoids long tail tasks and outperforms SparkGA in execution time.

**Table 7:** Comparison of execution time on D1 (unit: min)

Number of threads	1	8	16	24	32
GATK3.8 HaplotypeCaller	1372.33	369.77	356.21	361.19	326.48
ADS-HCSpark	1384.21	180.07	110.40	92.34	83.81

**Table 8:** Comparison of execution time on D1 (unit: min)

Number of threads	6	12	24	48	96	192
GATK4 HaplotypeCallerSpark	306.32	170.32	105.91	76.41	59.56	–
SparkGA	286.18	145.35	85.94	51.29	36.76	28.73
ADS-HCSpark	209.93	109.96	58.45	34.70	24.97	20.71

## Conclusion

In this paper, we present ADS-HCSpark, a scalable tool for variant calling based on Spark framework. ADS-HCSpark implements the parallelization of the mainstream variant calling algorithm HaplotypeCaller on multi-node and multi-core, accelerating the procession of variant calling. In ADS-HCSpark, a parallel strategy of adaptive data segmentation is proposed and a variant caller based on adaptive data segmentation (ADS-HC) is implemented to solve the problem of computation skew in HaplotypeCaller. Furthermore, for the requirement that adjacent data blocks should have overlapped boundaries, Hadoop-BAM library is customized to implement partitioning BAM file into overlapped blocks, improving the accuracy of variant calling. The performance of ADS-HCSpark is evaluated and the experimental result demonstrates that in the case of best performance that could be achieved in this experimental platform, ADS-HCSpark is 74% faster than GATK3.8 HaplotypeCaller on single-node experiments, 57% faster than GATK4.0 HaplotypeCallerSpark and 27% faster than SparkGA on multi-node experiments, with better scalability and the accuracy of over 99%. The future work will be to optimize performance and scale to large scale cloud computing platform.

## Availability and requirements

**Project name:** ASD-HCSpark

**Project home page:** http://github.com/SCUT-CCNL/ADS-HCSpark.git.

**Operating system:** Linux

**Programming language:** Java

**Other requirements:** Java 1.8, Scala 2.11.8, Hadoop 2.6.4, Spark2.2.0, Maven 3.5.3

**License:** New BSD License

**Any restrictions to use by non-academics:** none

## Additional files

Additional file 1: ADS-HCSpark’s source code. This is a compressed file and needs to be decompressed first. It contains all the code for this software. (ZIP 1793 kb)

Additional file 2: ADS-HCSpark’s usage document. This file introduces the software preparation environment and how to build and use ASD-HCSpark. (PDF 89 kb)

Additional file 3: The algorithm description of data preprocessing. This file includes the algorithm table and implementation details of data preprocessing. (PDF 47 kb)

Additional file 4: The algorithm description of computing the index number of data block to be split. This file includes the algorithm table and implementation details of computing the index number of data block to be split. (PDF 66 kb)

Additional file 5: The algorithm description of segmenting data blocks and sorting. This file includes the algorithm table and implementation details of segmenting data blocks and sorting. (PDF 51 kb)

Additional file 6: The algorithm description of acquiring overlapped data segments. This file includes the algorithm table and implementation details of acquiring overlapped data segments. (PDF 55 kb)

Additional file 7: The execution scripts. This file contains some execution scripts used in the experiments and some parameter settings. (PDF 64 kb)

Additional file 8: Dataset document. This file describes the datasets used in the experiments. (PDF 19 kb)

## Abbreviations

ADS-HC: a variant calling algorithm based on adaptive data segmentation
CPU: Central Processing Unit
DNA: Deoxyribonucleic acid
GATK: Genome Analysis Toolkit
GigE: Gigabit Ethernet
HDFS: Hadoop file system
I/O: input/output
NGS: next-generation sequencing
SNP: single nucleotide polymorphism
VCF: Variant Call Format
