# Supplementary material for: ADS-HCSpark: A scalable HaplotypeCaller leveraging adaptive data segmentation to accelerate variant calling on Spark
Source: BMC Bioinformatics. 2019 Feb 14;20:76. doi: 10.1186/s12859-019-2665-0 (PMC6376756; doi:10.1186/s12859-019-2665-0)
Supplement: Supplementary file 2 — ADS-HCSpark’s usage document. This file introduces the software preparation environment and how to build and use ASD-HCSpark. (PDF 89 kb) [file 12859_2019_2665_MOESM2_ESM.pdf]

ADS-HCSpark

ADS-HCSpark is a scalable tool to accelerate variant calling based on Apache Spark. It implements the parallelization of mainstream variant detection algorithm HaplotypeCaller on multi-node and multi-core. This file introduces the environment, build and usage of ADS-HCSpark, which contains the following sections.

1. System environment requirements
2. Build
3. Usage
4. Examples

System environment requirements

ADS-HCSpark is based on Apache Spark framework and GATK3.8 HaplotypeCaller, so some specific system environments and software packages are required. Note that ADS-HCSpark runs on a Spark cluster, so the cluster should have been properly setup. Maven is used to manage the project. Moveover, your system must be Linux-based.

- Java 1.8 or greater
- Scala 2.11.8 or greater
- Hadoop 2.6.4 or greater
- Spark 2.2.0 or greater
- Maven 3.5.3 or greater

Note that your Spark version should match the hadoop version. Each software package needs to be configured with relevant environment variables.

Bulid

ADS-HCSpark is based on the GATK3.8 HaplotypeCaller, so you should install GATK3 to the local Maven repository first.

1. Get the GATK3 code (or download the zip file directly)

   ```
   git clone https://github.com/broadgsa/gatk-protected.git
   ```

2. Go to the GATK3 folder and install it to local Maven repository. Note that the tag of gatk-protected.git should be 3.8. If not, please **git checkout 3.8** firstly.

   ```
   mvn install -P\!queue
   ```

3. Go to the ADS-HCSpark folder and build the code

   ```
   mvn package
   ```

4. After packaging, the executable jar package is `sparkhc-1.0-SNAPSHOT.jar` in the *target* folder.

Usage

ADS-HCSpark is implemented using a parallel strategy of adaptive data segmentation. It consists of two stages: Data preprocessing and ADS-HC. So, you should execute the data preprocessing program first and then execute variant caller ADS-HC. In ADS-HCSpark, there are three types of parameters. They are introduced below.

The first is Spark runtime parameters which need to be properly configured accoreding to the cluster. Note that ADS-HCSpark runs on Stand-alone mode. Theoretically, more cores and larger memory given, the better the performance. Since our program involves data segmentation, the parameters `spark.hadoop.mapreduce.input.fileinputformat.split.maxsize` and `spark.hadoop.mapreduce.input.file.fileinputformat.split.minsize` should be configured properly. Here we set them to 134217728. (The default data block size of the file on HDFS is 128MB on our cluster)

The second is specific parameters of ADS-HCSpark. They are explained as follows.

- **-t** : the tool name of executing. There are three tools: *BuildPreprocess*, *HaplotypeCaller*, and *vcfmerge*.
- **-i** : the input file path. The input file path should be an HDFS directory.
- **-o** : the ouput file path. It should be an HDFS directory. If this parameter is not specified, the outpu file path is the same as the input file path by default.
- **-p** : it represents using the adaptaive data segmentation. if this parameter is set, data preprocessing step need to be perfomed firstly.
- **-pf** : the preprocessing file path. When executing ADS-HC, configuring this parameter to indicate the path of preprocessing file. If it is not specified, the default preprocessing file path is the same as the input file path. The name of preprocessing file is the input file name plus the ".hcidx" suffix.
- **-s** : sorting and merging into a VCF file. When the merged file is too large, the parameter `spark.dirver.maxResultSize` should be adjusted appropriately.
- **-dc** : Cache optimization strategy. It is recommended to use when dbSNP is need.
- **-c** : specify a property file.

The third is other configuration option in the property file which is specified by using the parameter *-c* in the execution script. Here is an example of property file.

```
FASTA_PREFIX=/path/ucsc.hg19
DBSNP_DB=none
ADDITION_EACH_SPLIT_SIZE=4
```

- **FASTA_PREFIX** : This is the path prefix of the Reference file. The reference files such as "*.dict*", "*.fasta*", and "*.fasta.fai*" should be saved in this directory.
- **DBSNP_DB** : The dbSNP path. The value "none" means not to use dbSNP.
- **ADDITION_EACH_SPLIT_SIZE** : The granularity of data segmentation. In adaptive data segmentation, when time-consuming data blocks are found, the number of chunks that they wiil be segmented into is determined by this parameter.

Examples

The following are some examples of executaion scripts.

Data Preprocessing

```
./spark-submit \
--master spark://master:7077 \
--total-executor-cores 32 \
--driver-memory 50G \
--executor-memory 50G \
--conf spark.hadoop.mapreduce.input.fileinputformat.split.maxsize=134217728 \
--conf spark.hadoop.mapreduce.input.fileinputformat.split.minsize=134217728 \
/path/sparkhc-1.0-SNAPSHOT.jar \
-t BuildPreprocess \
-i /path/ERR091571.bam \
-c /path/conf.prop
```

ADS-HC

```
./spark-submit \
--master spark://master:7077 \
--total-executor-cores 32 \
--driver-memory 50G \
--executor-memory 50G \
--conf spark.hadoop.mapreduce.input.fileinputformat.split.maxsize=134217728 \
--conf spark.hadoop.mapreduce.input.fileinputformat.split.minsize=134217728 \
/path/sparkhc-1.0-SNAPSHOT.jar \
-t HaplotypeCaller \
-i /path/ERR091571.bam \
-o /path/ERR091671.vcf \
-c /path/conf.prop \
-p
```

Merge VCF

```
./spark-submit \
--master local[*] \
--driver-memory 50G \
--conf spark.hadoop.mapreduce.input.fileinputformat.split.maxsize=134217728 \
--conf spark.hadoop.mapreduce.input.fileinputformat.split.minsize=134217728 \
/path/sparkhc-1.0-SNAPSHOT.jar \
-t vcfmerge
-i ERR091571-folder
-o ERR091571.vcf
```
