# Supplementary material for: ADS-HCSpark: A scalable HaplotypeCaller leveraging adaptive data segmentation to accelerate variant calling on Spark
Source: BMC Bioinformatics. 2019 Feb 14;20:76. doi: 10.1186/s12859-019-2665-0 (PMC6376756; doi:10.1186/s12859-019-2665-0)
Supplement: Supplementary file 3 — The algorithm description of data preprocessing. This file includes the algorithm table and implementation details of data preprocessing. (PDF 47 kb) [file 12859_2019_2665_MOESM3_ESM.pdf]

Data preprocessing

Algorithm 1: Scanning and getting sequence features

```
Input:  BAM file
Output: [ indexID, sequencingFeature ]
Upload input BAM file to HDFS
samRdd = readHadoopBAMfile(BAM file)
filteredRdd = samRdd.filter ( x -> HaplotypeCallerFilter(x) )
[indexID, sequencingFeatures] = filterRdd.mapPartitionWithIndex(
      return getInterval () + getRecordNum () + getCIGAR_I () + getCIGAR_R ()
   )
return [indexID, sequencingFeature]
```

The algorithm description for scanning and getting sequence features is shown as algorithm 1. The input BAM file should be uploaded to HDFS where the BAM file is partitioned into several fix size data blocks (eg.128 MB) by default. Since ADS-HCSpark is closely related to HaplotypeCaller, it is necessary to attach the filter of HaplotypeCaller. ADS-HCSpark processes every data block through the operator *mapPartitionWithIndex* in Spark. Different from another operator *mapPartition* in Spark, *mapPartitionWithIndex* enables us to obtain the index number of the processed data block, so that the sequence features can corresponded to the data block for subsequent processing. ADS-HCSpark reads each block in parallel and counts sequence features of each block according to the corresponding field of every record in the block. Among sequence features, Interval and RecordNum can be obtained by separately counting the number of bases and the number of records in the data block. CIGAR_I and CIGAR_R could be calculated from the field CIGAR of every record in the block. Finally, Spark Driver program collects sequence features of all the blocks and saves them into a text file.
