# Supplementary material for: ADS-HCSpark: A scalable HaplotypeCaller leveraging adaptive data segmentation to accelerate variant calling on Spark
Source: BMC Bioinformatics. 2019 Feb 14;20:76. doi: 10.1186/s12859-019-2665-0 (PMC6376756; doi:10.1186/s12859-019-2665-0)
Supplement: Supplementary file 4 — The algorithm description of computing the index number of data block to be split. This file includes the algorithm table and implementation details of computing the index number of data block to be split. (PDF 66 kb) [file 12859_2019_2665_MOESM4_ESM.pdf]

Algorithm 2: Computing the index number of blocks to be split

```
Input:  PreprocessEntities
Output: PrioritySet
1:  prioritySet ← an empty HashSet
2:  IntervalLow2HighPercent ← ⌈preprocessEntities.size * m%⌉
3:  IntervalHigh2LowPercent ← ⌈preprocessEntities.size * k%⌉
4:  RecordNumPercent ← ⌈preprocessEntities.size * s%⌉
5:  CIGARPercent ← ⌈preprocessEntities.size * r%⌉
6:  preprocessEntities are sorted by Interval from low to high
7:  for i = 0; i < IntervalLow2HighPercent; i++ do
8:      prioritySet.add(preprocessEntities.get(i)
9:  end for
10: preprocessEntities are sorted by Interval from high to low
11: for i = 0; i < IntervalHigh2LowPercent; i++ do
12:     prioritySet.add(preprocessEntities.get(i)
13: end for
14: preprocessEntities are sorted by RecordNum from low to high
15: for i = 0; i < RecordNumPercent; i++ do
16:     prioritySet.add(preprocessEntities.get(i)
17: end for
18: preprocessEntities are sorted by (CIGAR_I+CIGAR_D) from high to low
19: for i = 0; i < CIGARPercent; i++ do
20:     prioritySet.add(preprocessEntities.get(i)
21: end for
22: return prioritySet
```

The algorithm description for computing the index number of data block to be split according above rules is shown as algorithm 2. The input *PreprocessEntities* is the preprocessing result, which includes the index of every original data blocks and the corresponding sequence features, and the output *prioritySet* is a HashSet to store the index number of blocks to be split. At the beginning of Algorithm 2, the number of data blocks required by each rule is calculated (line 2-5 in the Algorithm 2). Then the *preprocessEntities* is sorted by four rules mentioned above and the index of data block that satisfies the conditions will be added to the *prioritySet*. *PrioritySet* is a HashSet, a collection that does not

store duplicate elements. Some data blocks may satisfy two or more rules at the same time, so HashSet is used to exclude duplicate elements and besides, it could achieve O (1) time complexity of reading and storing.
