# Supplementary material for: ADS-HCSpark: A scalable HaplotypeCaller leveraging adaptive data segmentation to accelerate variant calling on Spark
Source: BMC Bioinformatics. 2019 Feb 14;20:76. doi: 10.1186/s12859-019-2665-0 (PMC6376756; doi:10.1186/s12859-019-2665-0)
Supplement: Supplementary file 5 — The algorithm description of segmenting data blocks and sorting. This file includes the algorithm table and implementation details of segmenting data blocks and sorting. (PDF 51 kb) [file 12859_2019_2665_MOESM5_ESM.pdf]

Algorithm 3: Segmentation and sorting of data blocks

```
Input:  splits, prioritySet
Output: prioritySplits
prioritySplits ← an empty list
for i = 0; i < splits.size; i++ do
    if prioritySet.contain(i) then
        prioritySplits.addAll(getSmall(splits.get(i)), HIGH_LEVEL)
    else
        prioritySplits.addAll(splits.get(i), NORMAL_LEVEL)
    end if
end for
prioritySplits are sorted by priority from high to low
return prioritySplits
```

The algorithm description for segmenting data blocks and sorting is shown as algorithm 3. The original data blocks are traversed sequentially and if the index number of the block exists in the *prioritySet*, it is finely divided into N new blocks which are set to high priority. The rest that are not in the *prioritySet* are not segmented and set to standard priority. After traversing all the data blocks, they will be sorted by their priority, thereby ensuring that time-consuming blocks will be processed firstly.
