# Supplementary material for: ADS-HCSpark: A scalable HaplotypeCaller leveraging adaptive data segmentation to accelerate variant calling on Spark
Source: BMC Bioinformatics. 2019 Feb 14;20:76. doi: 10.1186/s12859-019-2665-0 (PMC6376756; doi:10.1186/s12859-019-2665-0)
Supplement: Supplementary file 6 — The algorithm description of acquiring overlapped data segments. This file includes the algorithm table and implementation details of acquiring overlapped data segments. (PDF 55 kb) [file 12859_2019_2665_MOESM6_ESM.pdf]

Algorithm 4: Partition of BAM file with overlapped blocks

```
Input:  overlapSize
Output: Splits
splits ← Get the data blocks
splits are sorted by their file path and number
for split in splits do
    if split is not the last block then
        split.end ← split.end + overlapSize
    end if
    split.start ← the position of the first BAM record in the split
    split.end ← the end position of the last BGZF block in the split
end for
return splits
```

The algorithm description for acquiring overlapped data segments is shown as algorithm 4. The input parameter is the size of the overlapped area (*overlapSize*), which will affect the result of the subsequent variant calling. The experiment is conducted to evaluate it in detail in the later chapter. In the Algorithm 2, the data block information of the BAM file is obtained firstly and then data blocks are sorted according to the file path and the block number to ensure that the last block is at the last position of the link list and that an out-of-bounds error is avoided. Then the program traverses all the data blocks and except for the last data block, the rest need to be extended the size of overlapped area. The BAM file is compressed based on the BGZF (Blocked GNU Zip Format), which consists of many BGZF blocks. For each data block, the start position of the first BAM record is identified by modulus operation on BGZF blocks and other redundant information. To guarantee the integrity of the end of the expanded block, the algorithm shifts it slightly to cover the complete BGZF block. The method of determining the location comes from Hadoop-BAM. When finishing the processing, the program returns all the overlapped blocks.
