# Supplementary material for: ADS-HCSpark: A scalable HaplotypeCaller leveraging adaptive data segmentation to accelerate variant calling on Spark
Source: BMC Bioinformatics. 2019 Feb 14;20:76. doi: 10.1186/s12859-019-2665-0 (PMC6376756; doi:10.1186/s12859-019-2665-0)
Supplement: Supplementary file 7 — The execution scripts. This file contains some execution scripts used in the experiments and some parameter settings. (PDF 64 kb) [file 12859_2019_2665_MOESM7_ESM.pdf]

Execution scripts

This file contains some execution scripts used in the experiments and the some parameter settings. Note that our experimental platform has 6 nodes. Each node is equipped with two E5-2670 CPU (2.6GHz, 8 physical cores, 16 logical cores) with 62 GB memory. The network is 1 GigE. The parameters need to be set according to the actual experimental platform.

ADS-HCSpark

The following example is the execution scripts of ADS-HCSpark that used on the cluster with 6 nodes, and each node is loaded with 16 threads. If you want to adjust the number of threads, just only change the parameter *--total-executor-cores*.

Data Preprocessing

```
./spark-submit \
--master spark://master:7077 \
--total-executor-cores 48 \
--driver-memory 50g \
--executor-memory 50g \
--conf spark.hadoop.mapreduce.input.fileinputformat.split.maxsize=134217728 \
--conf spark.hadoop.mapreduce.input.fileinputformat.split.minsize=134217728 \
/path/sparkhc-1.0-SNAPSHOT.jar \
-t BuildPreprocess \
-i /path/ERR091572.bam \
-c /path/conf.prop
```

ADS-HC

```
./spark-submit \
--master spark://master:7077 \
--total-executor-cores 96 \
--driver-memory 50G \
--executor-memory 50G \
--conf spark.hadoop.mapreduce.input.fileinputformat.split.maxsize=134217728 \
--conf spark.hadoop.mapreduce.input.fileinputformat.split.minsize=134217728 \
/path/sparkhc-1.0-SNAPSHOT.jar \
-t HaplotypeCaller \
-i /path/ERR091572.bam \
-o /path/ERR091572.vcf \
-c /path/conf.prop \
-p
```

conf.prop

```
FASTA_PREFIX=/path/hg19
DBSNP_DB=none
ADDITION_EACH_SPLIT_SIZE=6
```

In the case of single node, the running mode of Spark could be set to **local**, and the parameter *--total-executor-cores* need to be adjusted accord condition of the machine. As mentioned in the paper, the optimal number of threads for preprocessing stage is 8 in our experimental platform. As for ADS-HC, usually more threads will achieve a higher performance. The related scriptes are as follows.

Data preprocessing

```
./spark-submit \
--master local[8] \
--driver-memory 50g \
--executor-memory 50g \
--conf spark.hadoop.mapreduce.input.fileinputformat.split.maxsize=134217728 \
--conf spark.hadoop.mapreduce.input.fileinputformat.split.minsize=134217728 \
/path/sparkhc-1.0-SNAPSHOT.jar \
-t BuildPreprocess \
-i /path/ERR091572.bam \
-c /path/conf.prop
```

ADS-HC

```
./spark-submit \
--master local[16] \
--driver-memory 50G \
--executor-memory 50G \
--conf spark.hadoop.mapreduce.input.fileinputformat.split.maxsize=134217728 \
--conf spark.hadoop.mapreduce.input.fileinputformat.split.minsize=134217728 \
/path/sparkhc-1.0-SNAPSHOT.jar \
-t HaplotypeCaller \
-i /path/ERR091572.bam \
-o /path/ERR091572.vcf \
-c /path/conf.prop \
-p
```

conf.prop

```
FASTA_PREFIX=/path/hg19
DBSNP_DB=none
ADDITION_EACH_SPLIT_SIZE=1
```

GATK4 HaplotypeCallerSpark

The following example is the execution scripts of HaplotypeCallerSpark that used on the cluster with 6 nodes and each node is loaded with 16 threads.

```
./gatk HaplotypeCallerSpark \
-R /path/hg19.fasta.2bit \
-I /path/ERR091572.bam \
-O /path/ERR091572.vcf \
-- --spark-runner SPARK \
--spark-master spark://master:7077 \
--total-executor-cores 96 \
--executor-memory 50G \
--driver-memory 50G
```

HaplotypeCaller

The following example is the execution scripts of HaplotypeCaller that used on a single node with 16 threads.

```
java -jar GenomeAnalysisTK.jar \
-T HaplotypeCaller \
-R /path/hg19.fasta \
-I /path/ERR091572.bam \
-O /path/ERR091572.vcf \
-nct 32
```
