# Supplementary material for: ADS-HCSpark: A scalable HaplotypeCaller leveraging adaptive data segmentation to accelerate variant calling on Spark
Source: BMC Bioinformatics. 2019 Feb 14;20:76. doi: 10.1186/s12859-019-2665-0 (PMC6376756; doi:10.1186/s12859-019-2665-0)
Supplement: Supplementary file 8 — Dataset document. This file describes the datasets used in the experiments. (PDF 19 kb) [file 12859_2019_2665_MOESM8_ESM.pdf]

Datasets

This file is mainly a description of the datasets used in the experiments.

The datasets we used in the experiements are public human genome datasets which has been widely used in ralated research. They could be available from http://smash.cs.berkeley.edu.

In the paper, dataset D1 is the ERR091572 from NA12878, D2 is the ERR091571 + ERR091572 from NA12878, and D3 is the ERR091787 from NA18507.
